# Selective Femoral Resurfacing for Bilateral Knee Osteonecrosis: A Bone‐Preserving Alternative to Total Knee Arthroplasty

**DOI:** 10.1155/cro/9478427

**Published:** 2026-04-08

**Authors:** Luca Cevolani, Laura Campanacci, Eric Lodewijk Staals, Paolo Spinnato, Marco Manfrini, Roberta Laranga, Mark Spitoni, Giuseppe Bianchi

**Affiliations:** ^1^ Unit of 3rd Orthopedic and Traumatologic Clinic Prevalently Oncologic, IRCCS Istituto Ortopedico Rizzoli, Bologna, Italy, ior.it; ^2^ Diagnostic and Interventional Radiology, IRCCS Istituto Ortopedico Rizzoli, Bologna, Italy, ior.it; ^3^ Unit of Biostatistics, Epidemiology and Public Health, Department of Cardiac, Thoracic, Vascular Sciences and Public Health, University of Padova, Padova, Italy, unipd.it; ^4^ Department of Biomedical and Neuromotor Sciences (DIBINEM), University of Bologna, Bologna, Italy, unibo.it

**Keywords:** clinical outcome, distal femur resurfacing, knee endoprosthesis, knee osteonecrosis, secondary avascular necrosis

## Abstract

**Introduction:**

Symptomatic avascular necrosis of the knee in young patients represents a therapeutic challenge. We present two cases of patients with bilateral massive osteonecrosis and advanced femoral chondropathy treated with bilateral femoral resurfacing.

**Conclusion:**

Prosthetic resurfacing of the distal femur in symptomatic osteonecrosis may represent a bone‐sparing technique for young patients, specifically targeting the pathological compartments. It allows for a rapid return to normal activity, reduces pain, and may delay the need for total knee replacement.

## 1. Introduction

Osteonecrosis of the knee is a devastating and irreversible disease often leading to end‐stage arthritis. Although it represents the second most frequently involved anatomical location after the hip [1] and remains a relatively uncommon cause of severe knee pain, it has increasingly become a significant complication following treatment for pediatric hematologic malignancies [2, 3], prolonged corticosteroid use, or other recognized predisposing conditions such as alcohol abuse, obesity, chemotherapy, dialysis, organ transplantation, and various systemic diseases [1, 4–9].

The management approach to secondary osteonecrosis depends upon the stage of the disease, clinical manifestations, size and location of the lesions, unilateral or bilateral joint involvement, patient age, activity level, general health, and life expectancy.

Conservative treatments, employing lifestyle modifications, pharmacologic therapy, or hyperbaric oxygen, are generally recommended in early‐stage disease or for smaller lesions [10, 11]. Joint‐preserving surgical options are typically indicated in symptomatic patients who fail conservative management and exhibit no evidence of joint collapse. These procedures include cartilage‐stimulating techniques such as arthroscopic debridement, removal of loose bodies, treatment of meniscal and chondral lesions, marrow stimulation techniques including microfracture and abrasion arthroplasty, or procedures aimed at restoring subchondral bone integrity, such as core decompression and bone grafting [12–14].

In advanced stages characterized by joint collapse, total knee arthroplasty represents the main surgical approach available to provide symptomatic relief and restore the knee′s functional range of motion [15]. However, total knee arthroplasty in younger patients predictably leads to the necessity for at least one or more revision procedures, due in part to the increased functional demands typical of this patient population. To decrease the risk of premature prosthetic wear and to preserve as much healthy knee tissue as possible, we opted to perform a knee hemiarthroplasty.

In this report, we describe two cases of young patients who presented with bilateral distal femoral osteonecrosis secondary to chemotherapy and were treated with bilateral distal femoral resurfacing.

### 1.1. Case 1

An 18‐year‐old female patient was referred to our service for bilateral knee pain not related to previous trauma or surgery. She had been diagnosed with acute lymphoblastic leukemia 18 months earlier. When she was admitted to our department, the leukemia was in remission, and there was no evidence of systemic infection. The patient reported pain and weakness when walking. The physical examination revealed medial joint line tenderness in both knees. Active knee flexion was 110° on the left and 120° on the right, with pain at maximum flexion. Knee extension was complete bilaterally and pain‐free. Additionally, both knees were swollen, but no joint instability was noted. The Knee Society Score (KSS) was 48 for the left knee and 50 for the right knee.

Preoperative anteroposterior and lateral radiographs revealed bilateral secondary osteonecrosis of the femoral condyles, with osteophytic lipping documented at the tibial plateau. MRI showed severe joint effusion and multiple bicondylar osteonecrotic foci in the distal femora, associated with intramedullary osteonecrosis, without involvement of the tibial plateau (Figure 1).

Figure 1(a) Preoperative anteroposterior radiograph demonstrating extensive bicondylar osteonecrosis following steroid therapy. (b) Postoperative anteroposterior plain radiograph after distal femoral resurfacing of the right knee; (c) the left knee was treated 6 months later. (d) Anteroposterior radiograph at 10‐year follow‐up.

**Figure:**
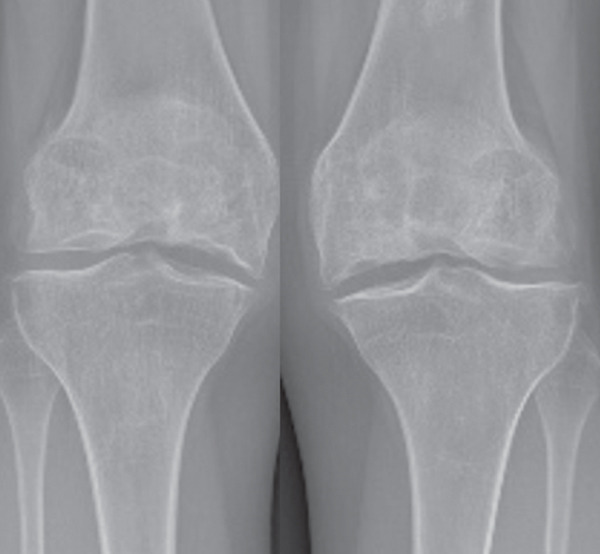
(a)

**Figure:**
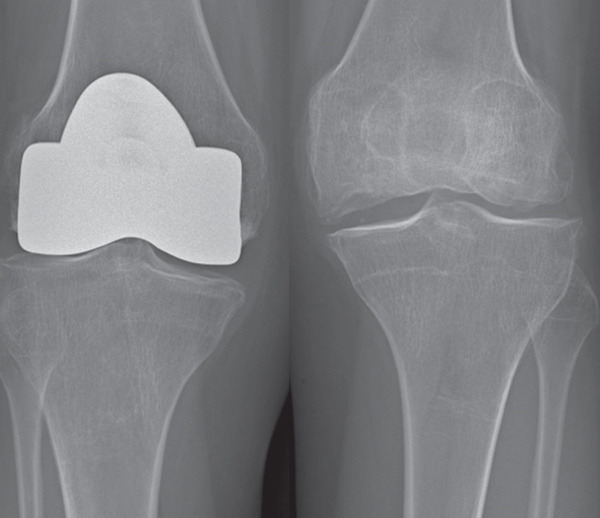
(b)

**Figure:**
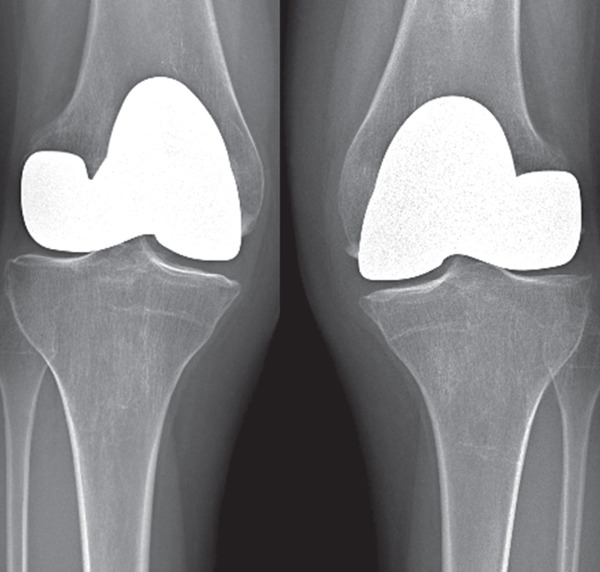
(c)

**Figure:**
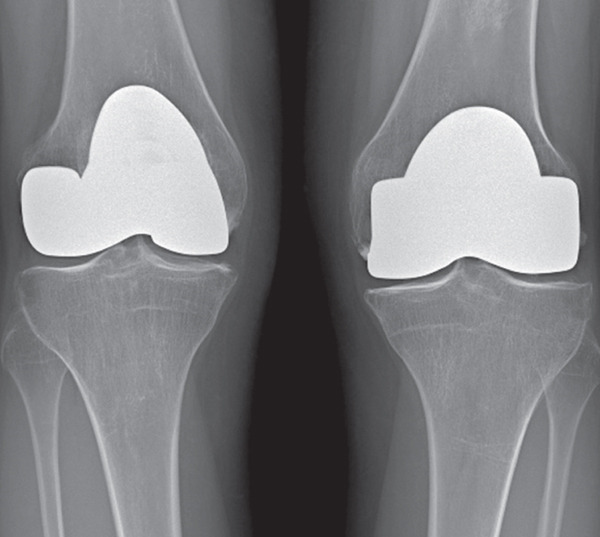
(d)

A partial hemiarthroplasty was initially performed on the right knee; the left knee was treated 6 months later. Intraoperative evaluation confirmed no sign of macroscopic ligament injury and almost normal menisci. Tibial plateau chondropathy Grade 1 according to the Outerbridge classification was detected in both knees. Normal physical activity was allowed 3 months after the final surgery. At follow‐up, no pain during ambulation was reported, and normal knee range of motion was observed, with 140° of active flexion in both knees.

Five years later, the patient returned to our clinic due to weight‐bearing medial pain in the left knee without a history of trauma. A knee arthroscopy was performed, showing joint arthrofibrosis, a medial meniscus body tear, and a Grade 2 Outerbridge tibial plateau chondropathy. A selective arthroscopic meniscectomy was performed, and the patient returned to normal physical activity 2 months after surgery.

At the 10‐year follow‐up, the KSS had improved to 87 and 96 for the left and right knee, respectively. The patient walked pain‐free and without assistance, with active flexion of 90° in the left knee and 120° in the right knee. Radiographs at the 10‐year follow‐up showed multiple tibial marginal osteophytes and tibial plateau osteochondral sinking bilaterally (Figure 1). No evidence of implant loosening, infection, or knee instability was detected, and no progressive radiolucent lines around the femoral components were observed. A knee prosthesis was deemed unnecessary, as the patient remained symptom‐free and in stable general condition at the time this manuscript was prepared.

### 1.2. Case 2

A 19‐year‐old male patient was referred to our service for bilateral knee pain 14 months after the end of high‐dose steroid treatment for acute lymphoblastic leukemia. When he was admitted to our department, the leukemia was in remission, and there was no evidence of systemic infection. Physical examination showed swollen knees; active flexion up to 130° and 120° for the left and right knee, respectively; and pain at maximum degrees of flexion without a sign of instability. The KSS was 53 and 57, respectively, for the right and left knee. CT scan showed intramedullary femoral necrosis with subchondral collapse in the right knee and medial condyle collapse in the left knee.

A partial hemiarthroplasty was initially performed on the right knee; the left knee was treated 6 months later. Intraoperative evaluation confirmed no sign of macroscopic ligament injury and almost normal menisci. Tibial plateau chondropathy Grade 1 according to the Outerbridge classification was detected in both knees (Figure 2). The patient resumed normal physical activity 3 months after the last surgery with complete pain control without drugs.

Figure 2(a) Intraoperative evaluation revealed advanced chondral damage of the distal femur, classified as Grade 4 according to the Outerbridge scale. (b) Femoral bone cuts were performed while preserving both the anterior and posterior cruciate ligaments. (c) At the end of the procedure, both cruciate ligaments and the native menisci were left intact.

**Figure:**
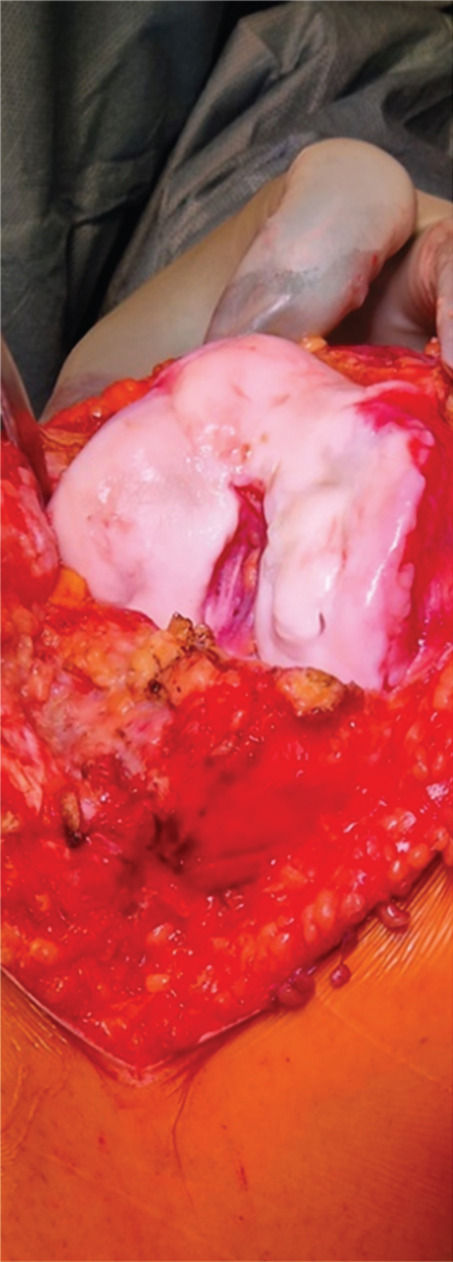
(a)

**Figure:**
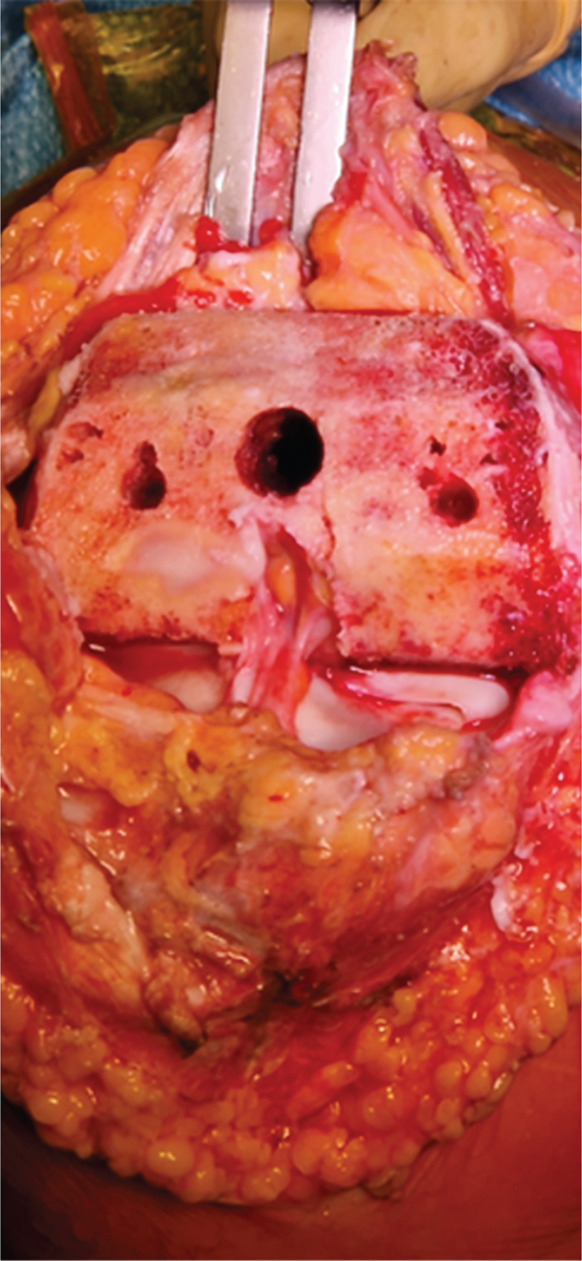
(b)

**Figure:**
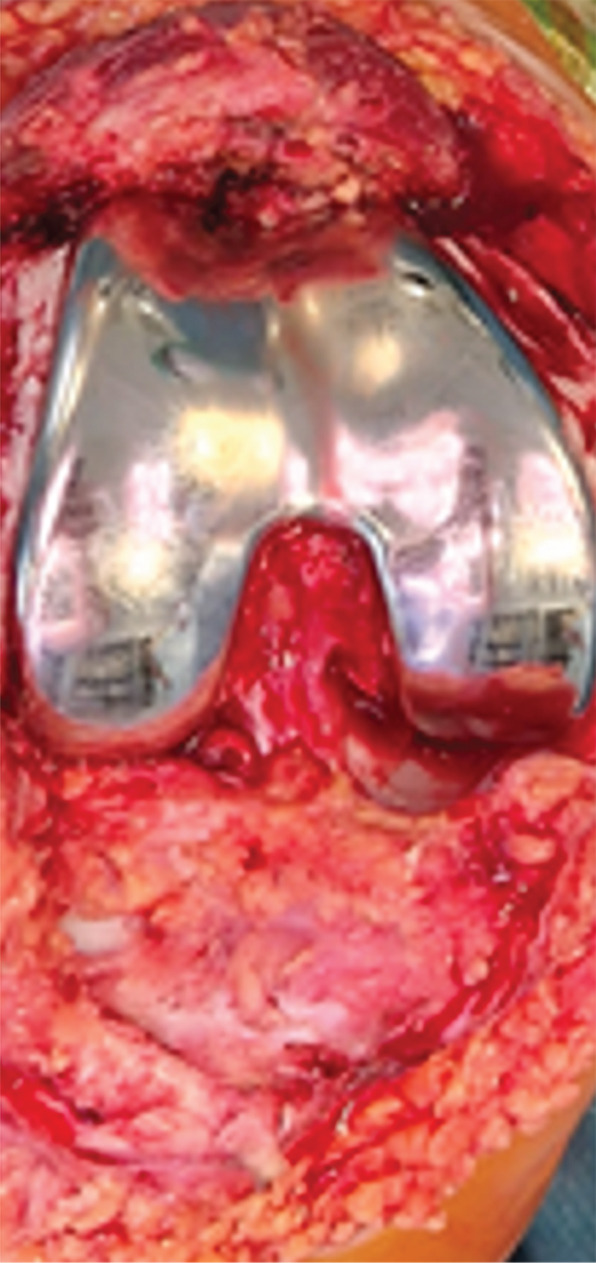
(c)

Intraoperative evaluation confirms no sign of macroscopic ligament lesions and normal menisci as well. Tibial plateau chondropathy Grade 1 according to the Outerbridge classification was detected on both knees.

Ten years later, there was no pain in both knees, and he could walk unaided. His active range of motion was 130° and 120° for the left and right knees, respectively. The KSS value was 96 and 100 for the left and right knees. The x‐ray showed multiple tibial osteophytes and tibial plateau osteochondral sinking, without signs of implant loosening (Figure 3). No indication for knee prosthesis was given, as the patient was asymptomatic and in good overall health at the time of writing this report.

Figure 3(a) Preoperative anteroposterior radiograph showing extensive diaphyseal osteonecrosis with associated subchondral collapse. (b, c) Radiographic images at 6 and 12 months following resurfacing arthroplasty of the right and left femur, respectively. (d) Plain radiograph at the 10‐year follow‐up showing early signs of knee osteoarthritis.

**Figure:**
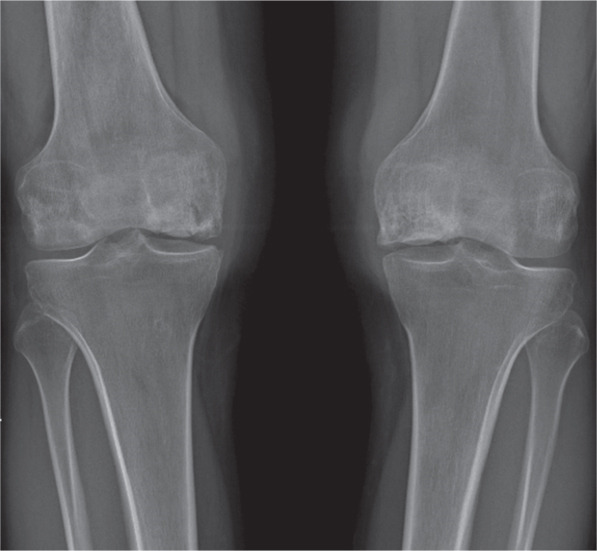
(a)

**Figure:**
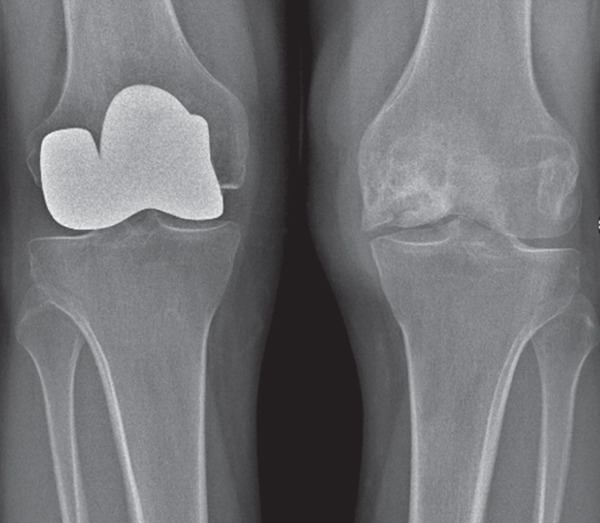
(b)

**Figure:**
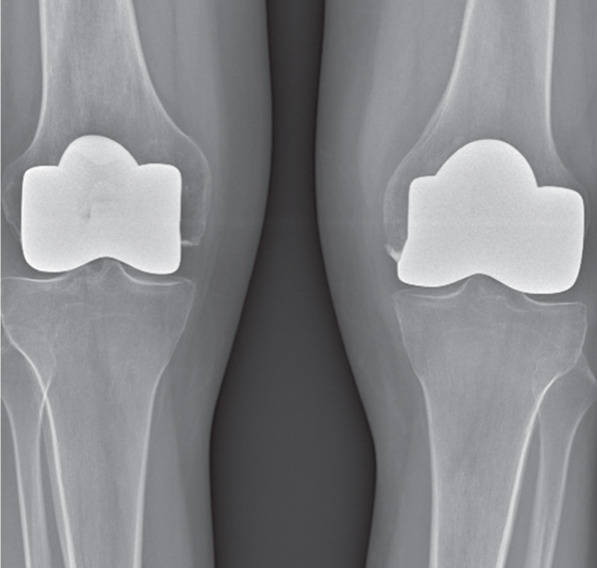
(c)

**Figure:**
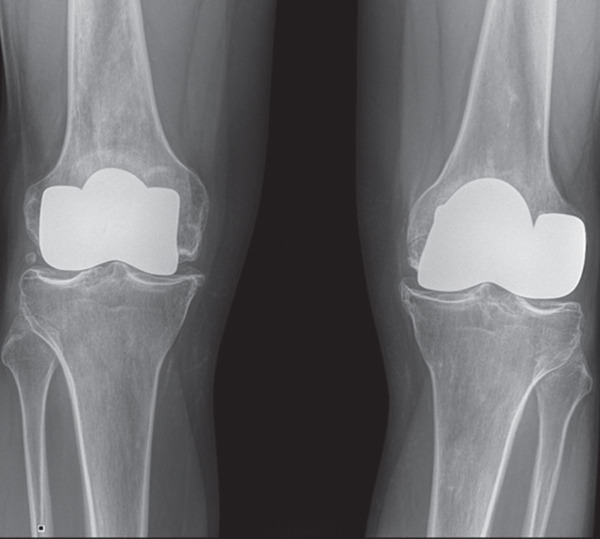
(d)

## 2. Rehabilitation

Both patients started the physiotherapy protocol on the first postoperative day with isometric quadriceps exercises, passive flexion (initially ranging from 0° to 30°), active knee mobilization, and progressive weight‐bearing mobilization using a walking frame or crutches as tolerated. Patients were advised to use a walking aid for at least 4 weeks and then to progress to full weight‐bearing as tolerated. All patients received prophylactic anticoagulation with low‐molecular‐weight heparin. Anticoagulation was discontinued when patients were able to fully weight‐bear.

## 3. Discussion

Total cemented or unicompartmental arthroplasty is considered the standard of care for advanced distal femur osteonecrosis. However, osteonecrosis secondary to steroid therapy for hematologic disease typically involves subchondral bone and the distal physis, and most cases are bicondylar. This represents a significant limitation of unicompartmental arthroplasty [16, 17].

Despite modern cemented prosthetic designs providing excellent functional results comparable to those reported for primary osteoarthritis [15], the very early age of onset in secondary forms exposes patients to a higher likelihood of revision surgery in adult age. Since no other biological options are available, distal femur endoprosthetic resurfacing could be an alternative surgical technique to total knee arthroplasty, offering less invasive surgery and preserving tibial bone stock in anticipation of potential future revision surgery.

In the literature, knee endoprosthetic resurfacing was originally proposed with good clinical results in children following primary tumor resection, using resurfacing allograft replacements for both the distal femur [18] and proximal tibia [19, 20], with the aim of preserving bone stock and physis for maintaining growth potential.

The choice of prosthetic implant must balance the need to preserve both cruciate ligaments to achieve joint stability and the requirement to match the native proximal tibia anatomy as closely as possible, accommodating a greater range of motion for active young patients and enhancing the natural anteroposterior rollback. According to our cases, distal femoral endoprosthesis provided long‐term pain relief and good functional outcomes. Indeed, all knees were clinically stable with a flexion range reaching at least 120°. Postoperative pain may result from progressive chondropathy, meniscal tear, and arthrofibrosis. In our series, arthroscopic surgery successfully alleviated symptoms without the need for implant revision.

The presence of devitalized bone with multiple osteonecrotic foci extending to the epiphyseal–metaphyseal region raises concerns regarding aseptic loosening, as reported in previous mid‐ to long‐term studies of total knee arthroplasty [15]. In contrast, recent studies utilizing new cemented total knee arthroplasty designs have shown excellent results without signs of loosening at midterm follow‐up [21, 22]. Our study aligns with the most recent literature, demonstrating reliable cemented femoral component fixation at the bone–implant interface, without progressive radiolucent lines observed at final follow‐up.

Preoperative meniscal integrity is essential, providing the only barrier between the femoral prosthetic component and the tibial plateau, thereby preventing abnormal loading and undesired degenerative joint changes.

Plain radiographs at an average follow‐up of 10 years revealed signs of osteoarthritis with progressive marginal osteophytes, osteochondral sinking of the tibial plateau, and progressive chondropathy due to stress from the femoral shield. On the other hand, proximal tibial deterioration should be considered an expected complication of distal femoral resurfacing, and revision surgery should be indicated when progressive osteoarthritis leads to decreased range of motion and persistent pain.

Despite the limitations of a small patient cohort and the retrospective nature of the study, the results are supported by a 10‐year follow‐up. Thus, we believe this technique represents a viable approach to delay total knee replacement in young patients.

## 4. Conclusion

Prosthetic resurfacing of the distal femur in symptomatic osteonecrosis may represent a bone‐sparing technique in young patients, specifically targeting pathological compartments and resulting in a rapid return to normal activity, reduced pain, and delayed total knee replacement. However, adequate mechanical stability under varus–valgus stress and good integrity of the cruciate ligaments are necessary prerequisites for this surgical approach.

## Ethics Statement

The ethics committee of the hospital approved the study.

## Consent

All the patients allowed personal data processing, and informed consent was obtained from all individual participants included in the study.

## Disclosure

The study was performed at the Rizzoli Orthopedic Institute, Via Pupilli 1, 40136 Bologna, Italy.

## Conflicts of Interest

The authors declare no conflicts of interest.

## Author Contributions

Study conception and design: Dr. Giuseppe Bianchi. Acquisition of data: Dr. Luca Cevolani. Analysis and interpretation of data: Dr. Roberta Laranga and Dr. Paolo Spinnato. Drafting of manuscript: Dr. Luca Cevolani. Critical revision: Dr. Giuseppe Bianchi, Dr. Marco Manfrini, Dr. Eric Lodewijk Staals, Dr. Laura Campanacci, and Mark Spitoni.

## Funding

No funding was received for this research.

## Data Availability

The data that support the findings of this study are available on request from the corresponding author. The data are not publicly available due to privacy or ethical restrictions.

## References

[1] Mont M. A. , Baumgarten K. M. , RifaI A. , Bluemke D. A. , Jones L. C. , and Hungerford D. S. , Atraumatic Osteonecrosis of the Knee∗, JBJS. (2000) 82, no. 9, 1279–1290, 10.2106/00004623-200009000-00008, 2-s2.0-0033814121, 11005519. 11005519

[2] Karimova E. J. , Wozniak A. , Wu J. , Neel M. D. , and Kaste S. C. , How Does Osteonecrosis About the Knee Progress in Young Patients With Leukemia?: A 2- to 7-Year Study, Clinical Orthopaedics and Related Research. (2010) 468, no. 9, 2454–2459, 10.1007/s11999-010-1358-9, 2-s2.0-77955568471, 20582497. 20582497 PMC2919885

[3] Lemonne N. , Lamarre Y. , Romana M. , Mukisi-Mukaza M. , Hardy-Dessources M. D. , Tarer V. , Mougenel D. , Waltz X. , Tressières B. , Lalanne-Mistrih M. L. , Etienne-Julan M. , and Connes P. , Does Increased Red Blood Cell Deformability Raise the Risk for Osteonecrosis in Sickle Cell Anemia?, Blood. (2013) 121, no. 15, 3054–3056, 10.1182/blood-2013-01-480277, 2-s2.0-84879448228, 23580637. 23580637 PMC3988032

[4] Chang C. , Greenspan A. , and Gershwin M. E. , The Pathogenesis, Diagnosis and Clinical Manifestations of Steroid-Induced Osteonecrosis, Journal of Autoimmunity. (2020) 110, 102460, 10.1016/j.jaut.2020.102460, 32307211. 32307211

[5] Karim A. R. , Cherian J. J. , Jauregui J. J. , Pierce T. , and Mont M. A. , Osteonecrosis of the Knee: Review, Annals of Translational Medicine. (2015) 3, no. 1, 10.3978/j.issn.2305-5839.2014.11.13, 2-s2.0-85015506937, 25705638. PMC4293480 25705638

[6] Boontanapibul K. , Steere J. T. , Amanatullah D. F. , Huddleston J. I. , Maloney W. J. , and Goodman S. B. , Initial Presentation and Progression of Secondary Osteonecrosis of the Knee, Journal of Arthroplasty. (2020) 35, no. 10, 2798–2806, 10.1016/j.arth.2020.05.020, 32527695. 32527695

[7] Narváez J. , Narváez J. A. , Rodriguez-Moreno J. , and Roig-Escofet D. , Osteonecrosis of the Knee: Differences Among Idiopathic and Secondary Types, Rheumatology. (2000) 39, no. 9, 982–989, 10.1093/rheumatology/39.9.982, 2-s2.0-0033826668, 10986303. 10986303

[8] Fonseca F. , Osteochondral Allograft in a Patient with Avascular Necrosis of the Knee Secondary to Lupus, Revista Brasileira de Ortopedia. (2018) 53, no. 6, 797–801, 10.1016/j.rbo.2017.06.016, 2-s2.0-85035231181. 30377619 PMC6205016

[9] Marom N. , Koch J. E. J. , Beer Y. , Ellis M. , Ganot G. , Nyska M. , Maoz G. , and Hetsroni I. , Thrombophilia-Associated Factors in Patients With Spontaneous Osteonecrosis of the Knee, Cartilage. (2019) 10, no. 1, 53–60, 10.1177/1947603517749920, 2-s2.0-85061710367, 29308659. 29308659 PMC6376567

[10] Osmani F. , Thakkar S. , and Vigdorchik J. , The Utility of Conservative Treatment Modalities in the Management of Osteonecrosis, Bulletin/Hospital for Joint Diseases. (2017) 75, no. 3, 186–192. 28902603

[11] Agarawal J. P. , Swangsilpa T. , van der Linden Y. , Rades D. , Jeremic B. , and Hoskin P. J. , The Role of External Beam Radiotherapy in the Management of Bone Metastases, Clinical Oncology. (2006) 18, no. 10, 747–760, 10.1016/j.clon.2006.09.007, 2-s2.0-33750444930. 17168210

[12] Zmerly H. , Moscato M. , Akkawi I. , Galletti R. , and Di Gregori V. , Treatment Options for Secondary Osteonecrosis of the Knee, Orthopedic Reviews. (2022) 14, no. 2, 33639, 10.52965/001c.33639. 35775038 PMC9239350

[13] Goodman S. B. and Hwang K. L. , Treatment of Secondary Osteonecrosis of the Knee With Local Debridement and Osteoprogenitor Cell Grafting, Journal of Arthroplasty. (2015) 30, no. 11, 1892–1896, 10.1016/j.arth.2015.05.013, 2-s2.0-84948718600, 26067706. 26067706

[14] Flynn J. M. , Springfield D. S. , and Mankin H. J. , Osteoarticular Allografts to Treat Distal Femoral Osteonecrosis, Clinical Orthopaedics and Related Research. (1994) 303, 38–43, 10.1097/00003086-199406000-00006. 8194252

[15] Mont M. A. , Rifai A. , Baumgarten K. M. , Sheldon M. , and Hungerford D. S. , Total Knee Arthroplasty for Osteonecrosis, Journal of Bone & Joint Surgery. (2002) 84, no. 4, 599–603, 10.2106/00004623-200204000-00014, 2-s2.0-0036528805. 11940621

[16] Bruni D. , Iacono F. , Raspugli G. , Zaffagnini S. , and Marcacci M. , Is Unicompartmental Arthroplasty an Acceptable Option for Spontaneous Osteonecrosis of the Knee?, Clinical Orthopaedics and Related Research. (2012) 470, no. 5, 1442–1451, 10.1007/s11999-012-2246-2, 2-s2.0-84859838049, 22278850. 22278850 PMC3314777

[17] Parratte S. , Argenson J. N. A. , Dumas J. , and Aubaniac J. M. , Unicompartmental Knee Arthroplasty for Avascular Osteonecrosis, Clinical Orthopaedics and Related Research. (2007) 464, 37–42, 10.1097/BLO.0b013e31812f7821, 2-s2.0-35848933934. 17589365

[18] Errani C. , Tanzi P. , Ferra L. , Campanacci L. , Donati D. M. , and Manfrini M. , Resurfaced Allograft-Prosthetic Composite for Distal Femur Reconstruction in Children With Bone Tumor, European Journal of Orthopaedic Surgery & Traumatology. (2021) 31, no. 8, 1577–1582, 10.1007/s00590-021-02995-1, 34009472. 34009472

[19] Campanacci L. , Alì N. , Casanova J. M. P. S. , Kreshak J. , and Manfrini M. , Resurfaced Allograft-Prosthetic Composite for Proximal Tibial Reconstruction in Children: Intermediate-Term Results of an Original Technique, Journal of Bone and Joint Surgery American Volume. (2015) 97, no. 3, 241–250, 10.2106/JBJS.N.00447, 2-s2.0-84922342980, 25653325. 25653325

[20] Manfrini M. , Donati D. , Colangeli M. , and Campanacci L. , Resurfaced Allograft-Prosthetic Composite for Proximal Tibial Reconstruction in Children, JBJS Essential Surgical Techniques. (2016) 6, no. 1, 10.2106/JBJS.ST.15.00010, 2-s2.0-84977117445, 30237914. PMC6145618 30237914

[21] Chalmers B. P. , Mehrotra K. G. , Sierra R. J. , Pagnano M. W. , Taunton M. J. , and Abdel M. P. , Reliable Outcomes and Survivorship of Primary Total Knee Arthroplasty for Osteonecrosis of the Knee, Bone & Joint Journal. (2019) 101-B, no. 11, 1356–1361, 10.1302/0301-620X.101B11.BJJ-2019-0576.R1, 31674235. 31674235

[22] Boontanapibul K. , Amanatullah D. F. , Huddleston J. I. , Maloney W. J. , and Goodman S. B. , Outcomes of Cemented Total Knee Arthroplasty for Secondary Osteonecrosis of the Knee, Journal of Arthroplasty. (2021) 36, no. 2, 550–559, 10.1016/j.arth.2020.08.061, 33011011. 33011011

